# Extracellular volume fractions are not consistent in gray zones determined by late gadolinium enhancement imaging of myocardial infarction

**DOI:** 10.1186/1532-429X-18-S1-P95

**Published:** 2016-01-27

**Authors:** Paras Parikh, Jason Ng, Michael Markl, Timothy Carroll, Jeffrey J Goldberger, Brandon C Benefield, Justin Ng, Daniel C Lee

**Affiliations:** 1 Biomedical Engineering, Northwestern University, Chicago, OH USA; 2 Feinburg School of Medicine, Northwestern University, Chicago, IL USA

## Background

Patients with prior myocardial infarctions have a large amount injured myocardial tissue which becomes replaced with fibrotic tissue over time. In the periphery of this scar tissue resides tissue that is a mixture of normal myocardium and scar tissue which has anisotropic conduction properties. Late gadolinium enhancement imaging (LGE) has been used to delineate this area known as gray zone. The amount of gray zone has been shown to be associated with the relative arrhythmia risk of patients with prior myocardial infarction. The aim of the study is to determine whether a common gray zone characterization method identifies physiologically equivalent areas across different subjects. The extracellular volume fraction (ECV) is quantitative measurement of fibrosis density calculated using pre- and post-contrast T1 maps. We hypothesize that ECV measurements in LGE-determined gray zone will show significant inter-subject variability.

## Methods

Eight canines with chronic infarcts were evaluated 12-14 weeks after infarct creation using a 3D Phase Sensitive Inversion Recovery (3D-PSIR) Turbo FLASH sequence and a modified look-locker inversion recovery (MOLLI) sequence for T1 mapping prior to contrast injection and 15-20 minutes after contrast injection. A calibration curve relating signal intensity to T1 was created based on the same long axis slice in the 3D PSIR and the MOLLI. This was done using the T1 maps created before and after contrast was given for all eight dogs. Using previously published full width half maximum (FWHM) criteria, gray zone was separated from normal tissue and dense scar based on signal intensity values of the post-contrast 3D PSIR images. Pixel by pixel calculation of ECV was done to create 3D high resolution ECV maps using pre and post contrast T1 maps. Hematocrit was assumed to be 0.37 for all canines for ECV calculations. ECVs for dense scar, gray zone, and normal myocardial tissue were calculated and compared for all canines.

## Results

Mean ECV fractions, mean T1 values and mean signal intensities were analyzed for each of the eight canines. Two out of the nine canines did not show good contrast between blood pool and tissue in the 3D-PSIR images resulting in difficult analysis of infarct regions. Mean ECV values amongst the eight dogs showed distinct differences between dense scar, gray zones and normal myocardial tissue of 0.81 ± 0.13, 0.53 ± 0.07 and 0.34 ± 0.09, respectively. However, the plots in the figure show significant differences in gray zone, and dense scar ECV between the 8 animals (p < 0.0001 by ANOVA).

## Conclusions

ECV fractions for the canines were not consistently within specific ranges for gray zones. This suggests that LGE defined regions of gray zone using FWHM may not delineate physiologically consistent areas of myocardial tissue composition. Future studies are necessary to determine whether ECV analysis to identify peri-infarct regions would be a better predictor of arrhythmia risk.

**Figure 1:**
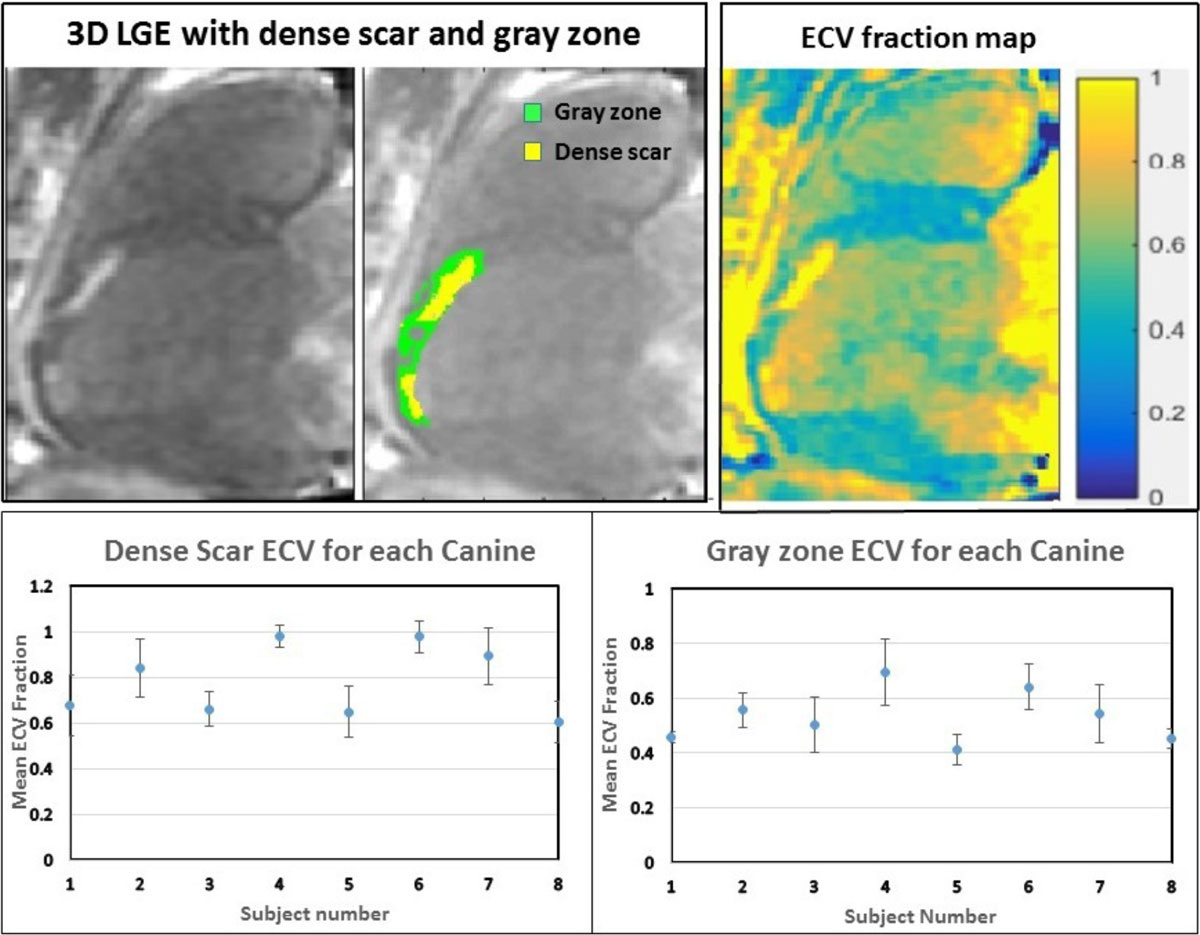
Figure 1

